# Role of Hypothalamic Creb-Binding Protein in Obesity and Molecular Reprogramming of Metabolic Substrates

**DOI:** 10.1371/journal.pone.0166381

**Published:** 2016-11-10

**Authors:** Cesar L. Moreno, Linda Yang, Penny A. Dacks, Fumiko Isoda, Jan M. A. van Deursen, Charles V. Mobbs

**Affiliations:** 1 Department of Neuroscience, and the Friedman Brain Institute, Icahn School of Medicine at Mount Sinai, New York, New York, United States of America; 2 Department of Pediatric and Adolescent Medicine, Mayo Clinic College of Medicine, Rochester, Minnesota, United States of America; Institut d'Investigacions Biomèdiques August Pi i Sunyer, SPAIN

## Abstract

We have reported a correlation between hypothalamic expression of Creb-binding protein (*Cbp*) and lifespan, and that inhibition of *Cbp* prevents protective effects of dietary restriction during aging, suggesting that hypothalamic *Cbp* plays a role in responses to nutritional status and energy balance. Recent GWAS and network analyses have also implicated *Cbp* as the most connected gene in protein-protein interactions in human Type 2 diabetes. The present studies address mechanisms mediating the role of *Cbp* in diabetes by inhibiting hypothalamic *Cbp* using a Cre-lox strategy. Inhibition of hypothalamic *Cbp* results in profound obesity and impaired glucose homeostasis, increased food intake, and decreased body temperature. In addition, these changes are accompanied by molecular evidence in the hypothalamus for impaired leptin and insulin signaling, a shift from glucose to lipid metabolism, and decreased *Pomc* mRNA, with no effect on locomotion. Further assessment of the significance of the metabolic switch demonstrated that enhanced expression of hypothalamic *Cpt1a*, which promotes lipid metabolism, similarly resulted in increased body weight and reduced *Pomc* mRNA.

## Introduction

The growing prevalence of type 2 diabetes associated with obesity constitutes one of the greatest threats to world health in the 21^st^ century [1], but mechanisms contributing to these syndromes remain to be elucidated. Nutrient-sensing hypothalamic neurons constitute a key locus for sensing and regulating energy balance and glucose metabolism, so mechanisms mediating these hypothalamic functions are of general concern. While hypothalamic sensing of leptin and insulin mediate aspects of energy and glucose homeostasis [2], specialized hypothalamic neurons also sense glucose by mechanisms similar to those of pancreatic beta cells [3], and these neurons can regulate blood glucose [4]. Hypothalamic glucose sensing neurons have long been hypothesized to play a role in regulating energy balance [5], but the role of these neurons in regulating energy balance remains to be determined. Nevertheless recent studies have suggested that a metabolic shift away from hypothalamic glucose metabolism and toward hypothalamic lipid metabolism promotes obese phenotypes [6,7].

We previously reported that hypothalamic expression of Creb-binding protein (*Cbp)* positively correlates with lifespan across 5 mouse strains [8]. More recently we have observed that dietary restriction induces hypothalamic *Cbp* in mice, associated with protective effects against proteotoxicity [9]. Similarly, dietary restriction in *Caenorhabditis elegans* induces *Cbp*, and blocking this induction prevents protective effects of dietary restriction during aging [8]. In addition, recent GWAS and network analyses have implicated *Cbp* as the most connected gene in protein-protein interactions in Type 2 diabetes [10]. These reports suggest a function for Cbp in nutrient sensing and energy balance.

The present studies examined the role of hypothalamic *Cbp* in energy balance and glucose homeostasis. These studies demonstrate that inhibition of hypothalamic *Cbp* produces robust obese phenotypes entailing both increased food intake and reduced body temperature, associated with impaired glucose homeostasis. These changes are associated with a hypothalamic molecular profile that indicates a shift away from glucose metabolism and toward lipid metabolism, even in pair-fed and weight-matched controls, supporting that this molecular profile causes, rather than is a result of, the obese phenotypes. Further supporting a causal role, we demonstrate that enhanced hypothalamic expression of carnitine palmitoyltransferase 1A (*Cpt1a*), a key enzyme promoting lipid metabolism, produces similar obese phenotypes, associated with reduced hypothalamic of hypothalamic Pro-opiomelanocorticotropin (*Pomc)*. These studies demonstrate that reduced hypothalamic *Cbp* promotes obese phenotypes, plausibly in part by enhancing hypothalamic lipid metabolism and reducing hypothalamic glucose metabolism, associated with reduced hypothalamic *Pomc*.

## Materials and Methods

### Animals

*Cbp*^(flox/flox)^ breeding pairs were received from the Jan van Deursen laboratory (Mayo Clinic, Rochester, MN); these were produced as described [11]. All mouse studies were specifically approved with permission and in accordance with the Institutional Animal Care and Use Committee (IACUC protocols 12–0044 and 12–0169) from the Icahn School of Medicine at Mount Sinai. Mice were kept under a 12-hour light: 12-hour dark cycles.

### Surgical procedures and viral delivery

*Cbp* inhibition by Cre recombinase viral delivery was carried out as described[12]. Surgeries were carried out in 8–10 week old mice. Animals were anesthetized using isoflurane and infused with adeno-associated virus expressing Cre-recombinase (AAV2/8.CMV.HI.eGFP-Cre.WPRE.SV40), or control (AAV2/8.CMV.PI.eGFP.WPRE.bGH). Viral serotypes AAV2/8 (Cre-recombinase or control) were purchased from Penn Vector Core at the University of Pennsylvania. AAV2/9.CPT1A and control (AAV2/9.mCherry) driven by the cytomegalovirus promoter were packaged by Virovek and Neurologix Inc., respectively. Two different serotypes of AAV were used due to availability of the respective vectors from their respective sources. An infusion cannula was introduced at 3mm/10s to target the mediobasal hypothalamus using coordinates: AP—1.5mm; ML ± .35mm; DV 6mm from bregma. One μl per hemisphere was infused (1μl/min), cannula was left to rest for 7 minutes and removed 1mm/min (first 2mm) and then 1mm/15s. After surgery animals were individually caged, where they were monitored and allowed to recover in warmed cages for up to 2 hours in order to prevent hypothermia. Animals were monitored daily and evaluated for any wound complications or infections for the duration of the experiments; any animals that lost more than 20% in body weights was sacrificed. Virus expression was corroborated by immunohistochemistry or RT-PCR where applicable.

### Immunohistochemistry

Animals were perfused using cold 4% paraformaldehyde in PBS as described[13]. Brain was extracted and placed in 4% paraformaldehyde at 4°C overnight. Free-floating sections were prepared by slicing at a 75μm thickness using a vibratome and collected in PBS. Brain slices were washed and blocked for 1.5 hours in 5% normal goat serum and 0.3% Triton X. Slices were incubated with Anti-CBP (SC-1211; 1:100) (Santa Cruz Biotechnology, Dallas, TX) O/N at 4°C. After incubation sections were stained with Alexa Fluor 658 Anti-rabbit IgG (A-11011; 1:400) and Hoescht (H1399; 2μg/ml) (Life Technologies). Sections were then mounted on slides using Fluoromount G (Beckman Coulter, Fullerton, CA). All images were acquired with a Zeiss LSM 780 confocal microscope using an 10x Neofluar objective. 1024x1024 12 bit images were captured using ZEN 2012 version 8.1, and processed in Fiji. CBP immunopositive cells from a single slide (75μm thickness) per animal that included the infusion site in the mediobasal hypothalamus using an ROI mask (S1 Fig) of the same area were counted and evaluated using a blinded design and the Analyze Particle feature in Fiji.

### Blood glucose and insulin measurements

Tail blood was collected and blood glucose was measured using a Bayer Contour glucose meter (Bayer, Mountain View, CA). Glucose tolerance tests were carried out after a 4-hour fast followed by an intraperitoneal (i.p) injection of 20% glucose in saline adapted to body weight (10μl/g). Blood insulin was obtained using ultra-sensitive mouse insulin ELISA kit (Crystal Chem, Downers Grove, IL).

### Food administration and animal records

All animals were individually caged after virus injections. Food intake was measured 2 weeks after viral delivery. Any spilled food was collected and subtracted from food intake measurements. Two control groups injected with either AAV-Cre or AAV-GFP were allowed free access to chow diet. Another two experimental groups were set up using the following protocol to match body weights from the time of surgery. The latter were weighed every day and given appropriate grams of food to maintain original body weight. To study energy expenditure starting one week after surgery, AAV-Cre or AAV-GFP infused groups were pair-fed by providing food pellets 1h before lights out. Animals received the NIH-31 chow formula (Harlan Teklad Laboratories, Madison, WI), or high fat diet 20% protein, 35% carbohydrate, 45% fat). Core body temperature was recorded after decapitation via rectal probe using IT-1E thermocouple microprobe and a Bat-12 thermometer (Physitemp, Clifton, NJ).

### Quantification of mRNA

**Fig 1:**
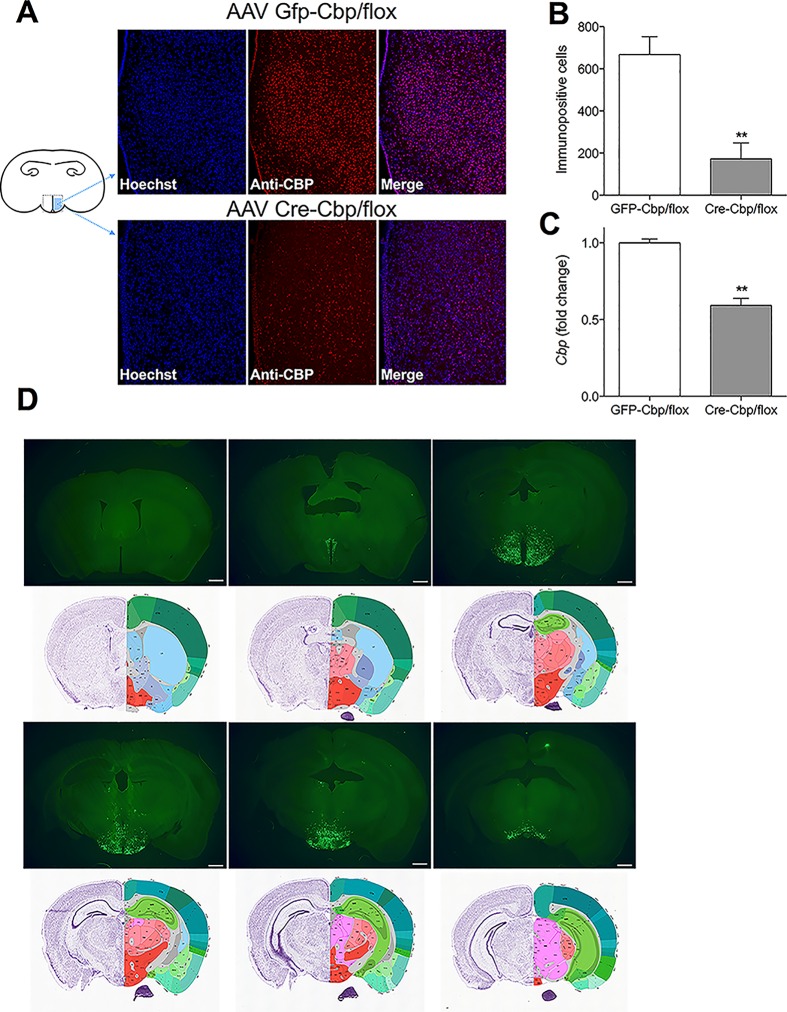
*Cbp*^flox/flox^ mice infused in the hypothalamus with AAV-Cre show reduced immunoreactivity and mRNA levels for *Cbp*. (A) Representative images showing nuclear Hoescht staining (blue), immunoreactive CBP (red) and colocalization (pink) after 3 weeks hypothalamic infusion of AAV-GFP or AAV-Cre (B) Number of immunopositive cells ± SEM in the mediobasal hypothalamus (*n* = 5–6) (C) Murine hypothalamic *Cbp* ± SEM quantified by RT-PCR (*n* = 14–20). (D) Representative images of coronal sections collected 3 weeks after stereotaxic delivery. Images are ordered from rostral to caudal along with corresponding Allen Atlas sections for reference [16]. Scale bar = 800 μm.

Animals were sacrificed by decapitation after a brief exposure to carbon dioxide. Brain tissue and dissections were performed in an ice-cold brain block as previously described[7]. Hypothalamic dissections included the area represented in pictures and diagram on Fig 1A. Tissue was frozen in dry ice and stored at -80°C. RNA was extracted using Qiazol reagent and miRNAeasy kit (Qiagen, Redwood City, CA). RNA was measured using a nanodrop ND-1000 spectrophotometer (Thermo Fisher Scientific, Wilmington, DE). cDNA was made using 500ng of RNA using RT2 First Strand Kit (SABiosciences, Frederick, MD). Data were gathered as Ct (threshold cycle) values obtained from ABI SDS software. Relative mRNA levels were determined by standard ΔΔCt methods and were expressed in fold change based on control AAV-GFP or AAV-mCherry animals when appropriate. 200ng of RNA were used in a custom-made nCounter Codeset (Nanostring Technologies, Seattle, WA) and were carried out in our facilities according to manufacturer protocols. Data was extracted using nSolver Analysis Software 1.1 (Nanostring Technologies, Seattle, WA) and values were normalized to positive controls and stable housekeeping transcripts (*Hprt*, *Hsp90ab1*, *Ppia*, *Rn18s*), which were not influenced by experimental conditions. Probes and RT-PCR primers utilized are listed in the S1 Table.

### Statistical analysis

All animal physiological records were analyzed using PRISM 5 Software using 2-way ANOVA followed by Bonferroni, or Student’s t-test where appropriate.

## Results

### Inhibition of hypothalamic *Cbp* in *Cbp*^flox/flox^ mice by AAV-Cre

To study the role of hypothalamic *Cbp* on energy balance, AAV-*Cre*-*recombinase* or control AAV-GFP was infused directed towards the ventromedial hypothalamus (as described [14]) (1μl per side) of *Cbp*^flox/flox^ mice (loxp flanking sites in exon 9 of the *Cbp* gene[11]). The AAV2/8 serotype was chosen based on publications assessing efficacy and stable expression in other brain areas[15]. Mice were infused and housed singly on a standard chow diet (NIH-31). Three weeks after infusion, this protocol resulted in a significant reduction of hypothalamic *Cbp* mRNA levels (41%) and CBP-immunopositive cells (74%) as indicated by immunohistochemistry using a CBP-specific antibody (Fig 1A–1C). In contrast cell numbers and morphology based on Hoescht staining, total RNA levels, and *Sf1* mRNA levels were not affected by the infusions, demonstrating that inhibition of *Cbp* did not cause loss of hypothalamic neurons (S2 Fig), consistent with previous studies[12]. Targeting of the virus towards the mediobasal hypothalamus resulted in robust reduction of *Cbp* mRNA and immunoreactivity (Fig 1A–1C), but this was also accompanied by noticeable spread of the virus particularly rostrally and caudally (Fig 1D), including nuclei in anterior sections of the paraventricular and the medial mammillary nucleus. Additionally, within the same plane of the coronal injections diffused lateral expression in the lateral, tuberal, dorsomedial nuclei was observed.

#### Inhibition of hypothalamic *Cbp* increases body weight and food intake and impairs glucose homeostasis

**Fig 2:**
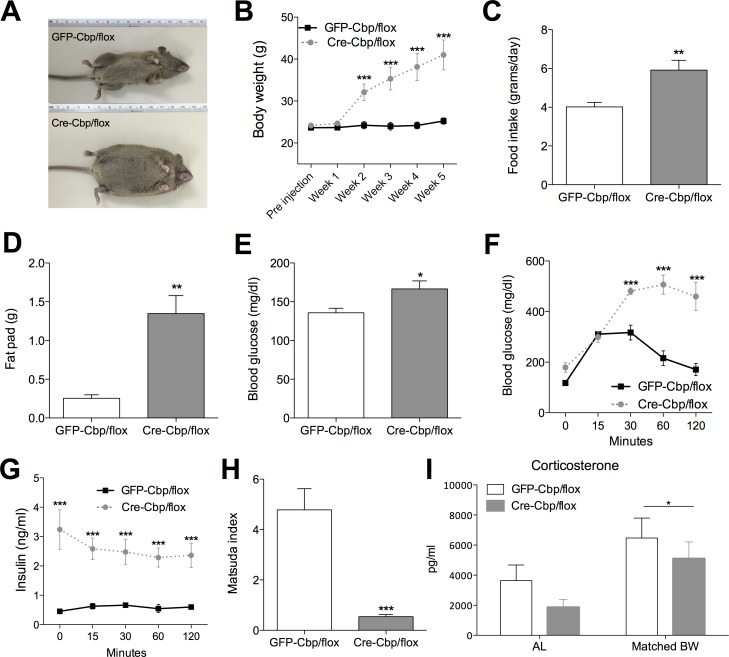
*Cbp*^flox/flox^ mice infused in the hypothalamus develop an obese phenotype, and associated metabolic impairments, compared to mice infused with AAV-GFP. (A) Representative physical appearance of control and CBP-inhibited littermates 5 weeks after infusions. (B) Ad libitum body weight ± SEM on a chow diet (*n* = 7–8), solid lines = GFP-Cbp/flox (controls); white bars = Cre-Cbp/flox (experimental). (C) Food intake ± SEM at 2 weeks after virus infusions (*n* = 7–8). (D) Visceral fat pad weights ± SEM 5 weeks after virus infusions (*n* = 8–9). (E) Blood glucose ± SEM at 2 weeks after infusions (*n* = 15–16). (F) Blood glucose and (G) insulin ± SEM during glucose tolerance tests at 3 weeks after virus infusions (*n* = 6). Solid lines = GFP-Cbp/flox (controls); white bars = Cre-Cbp/flox (experimental). (H) Whole body insulin sensitivity ± SEM as measured by Matsuda index at 3 weeks after virus injection (*n* = 6). (I) Corticosterone levels measured 3 weeks after virus infusions in Ad lib and body weight matched groups (n = 9–7). *, p < .05, **, p < .01. ***, p < .001.

The first week after infusion there were no significant changes in metabolic phenotypes, consistent with the property of AAV to maximally express about 2 weeks after delivery. Starting at two weeks after infusion, body weights significantly increased in Cre-Cbp/flox mice vs. GFP-Cbp/flox controls (Fig 2A and 2B), associated with increased food intake (Fig 2C) and increased adiposity as indicated by increased fat pad weight (Fig 2D). These robust and early increases in body weight were accompanied by increased baseline blood glucose, detectable by 2 weeks after infusion (Fig 2E). Similarly, plasma glucose was elevated during glucose tolerance tests in the Cre-Cbp/flox mice 30–120 minutes after a 4-hour fast followed by a weight-adjusted i.p. injection of glucose (Fig 2F). Strikingly, Cre-Cbp/flox mice were hyperinsulinemic throughout the glucose tolerance protocol (Fig 2G), reflecting whole-body insulin resistance as indicated by the computed Matsuda index (Fig 2H) [17]. That insulin levels did not change during the glucose tolerance test may indicate a ceiling effect, i.e., extreme insulin resistance leads to maximal insulin secretion, which is unresponsive to further glucose stimulation. Impairments in glucose homeostasis were not due to elevated corticosterone levels, which were not affected by inhibition of hypothalamic *Cbp* (Fig 2I), although the matched body weight group exhibited significantly higher levels of corticosterone as expected.

### Inhibition of hypothalamic *Cbp* decreases body temperature

**Fig 3:**
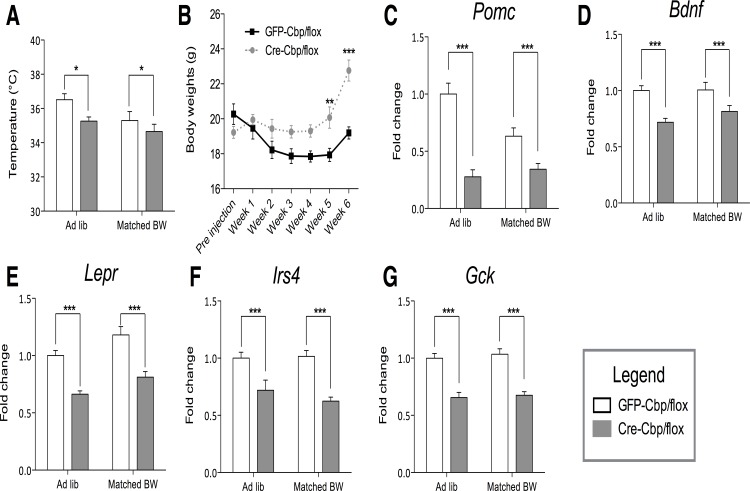
Inhibition of hypothalamic *Cbp* reduces body temperature, while impairing neuropeptide signaling. (A) Body temperature ± SEM of control and knockdown mice fed ad libitum (AL) chow diet or restricted diet to match body weights (Matched BW) (*n* = 5–10). (B) Body weights ± SEM of control and knockdown mice fed a fix diet below ad libitum food intake for 6 weeks after virus infusion. Solid lines = GFP-Cbp/flox (controls); white bars = Cre-Cbp/flox (experimental). Hypothalamic relative transcript abundances ± SEM as measured by nCounter, values are normalized to ad libitum control. White bars = GFP-Cbp/flox (controls); Solid bars = Cre-Cbp/flox (experimental). Murine genes (C) *Pomc*, (D) *Bdnf*, (E) *Lepr*, (F) *Irs4*, and (G) *Gck (n =* 8). *, p < .05. **, p < .01. ***, p < .001 by 2-Way ANOVA.

Obese phenotypes are associated with decreased core body temperatures, which plausibly indicate a decrease in energy expenditure [18,19]. For example, we have reported that body temperature is reduced in genetically obese *ob*/*ob* mice, and this is reversed by transgenic restoration of central POMC [20]. Inhibition of hypothalamic *Cbp* significantly reduced body temperature even in food-restricted animals matched to control for body weight (Fig 3A). To further assess if *Cbp* reduction produces obesity through decreased energy expenditure, we pair-fed Cre-Cbp/flox mice to match control ad lib food intake for 6 weeks. These mice were monitored daily to ensure that food was completely consumed. After 5 weeks, body weights of the Cre-Cbp/flox mice were statistically elevated relative to controls (Fig 3B), indicating hypothalamic *Cbp* may regulate energy balance through both food intake and energy expenditure.

### Inhibition of hypothalamic *Cbp* reduces hypothalamic expression of *Pomc* and *Bdnf*

The hypothalamus is particularly sensitive to metabolic state compared to other brain areas, and hypothalamic gene expression, particularly hypothalamic *Pomc*, is especially important in the regulation of energy balance and glucose homeostasis [7,20]. We therefore characterized the effect of inhibiting hypothalamic *Cbp* on hypothalamic gene expression, using a custom-designed nCounter assay allowing for highly sensitive and reproducible transcript counts without an amplification step [21]. A heat map was generated from this analysis (S3 Fig).

Inhibition of hypothalamic *Cbp* reduced expression of hypothalamic *Pomc* (Fig 3C) as well as *Bdnf* (Fig 3D) and other gene expression persisted even when body weights were matched by caloric restriction below pair-fed consumption. Reduced expression of both of these genes has been demonstrated to cause obesity and impaired glucose homeostasis, and at least partially mediates obese phenotypes in leptin-deficient mice [20,22]. Reduction in hypothalamic *Pomc* and *Bdnf* was consistent with reductions in the expression of leptin receptor (*Lepr*), insulin signaling (*Irs4*), and glucokinase (*Gck*) (Fig 3E–3G), all of which mediate hypothalamic responses to nutritional state [23,24]. Expression of other genes implicated in energy balance, like *Agrp*, were not influenced by reducing hypothalamic *Cbp*, while there was a modest decrease in *Npy* (data not shown).

### Inhibition of hypothalamic *Cbp* produces a transcriptional profile indicating decreased glucose metabolism and increased lipid metabolism

**Fig 4:**
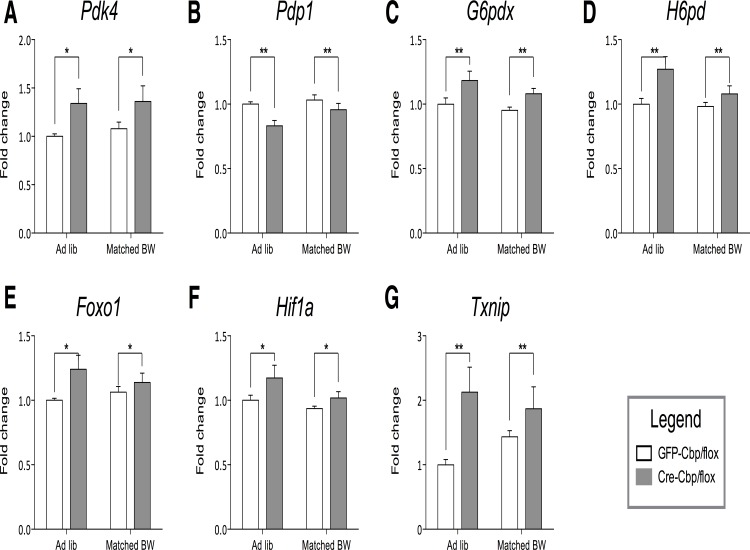
*Cbp* inhibition decreases hypothalamic glycolytic markers. Relative murine transcript abundances ± SEM as measured by nCounter, values are normalized to ad libitum control. White bars = GFP-Cbp/flox (controls); Solid bars = Cre-Cbp/flox (experimental). Murine genes (A) *Pdk4*, (B) *Pdp1*, (C) *G6pdx*, (D) *H6pd*, (E) *Foxo1*, (F) *Hif1a*, and (G) *Txnip (n =* 8). *, p < .05, **, p < .01. by 2-Way ANOVA.

**Fig 5:**
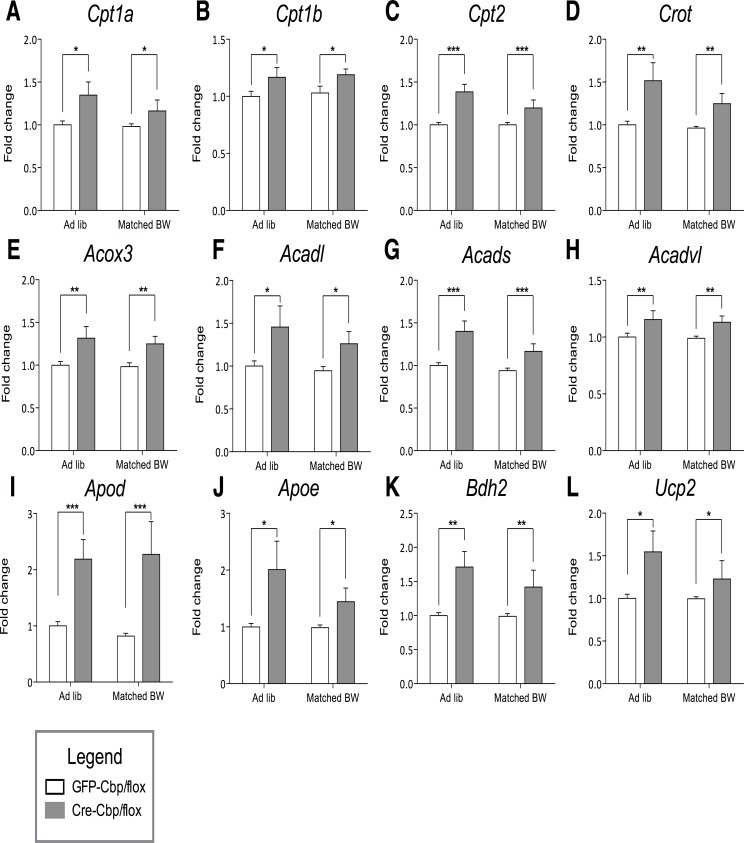
Obesity and *Cbp* knockdown show increased lipid and ketone oxidation markers in the hypothalamus. Relative murine transcript abundances ± SEM as measured by nCounter, values are normalized to ad libitum control. White bars = GFP-Cbp/flox (controls); Solid bars = Cre-Cbp/flox (experimental). Murine genes (A) *Cpt1a*, (B) *Cpt1b*, (C) *Cpt2*, (D) *Crot*, (E) *Acox3*, (F) *Acadl*, (G) *Acads*, (H) *Acadvl*, (I) *Apod*, (J) *Apoe*, (K) *Bdh2*, and (L) *Ucp2* (*n* = 8). *, p < .05. **, p < .01. ***, p < .001 by 2-Way ANOVA.

We have reported that fasting produces a hypothalamic gene expression profile indicating a shift from glycolysis toward lipid metabolism and alternative pathways for glucose metabolism [7]. Consistent with the observation that fasting-induced hypothalamic changes would be expected to produce obese phenotypes in the presence of food, inhibition of hypothalamic *Cbp* produced a similar hypothalamic shift away from glycolysis (and toward alternate pathways of glucose metabolism) and toward increased lipid metabolism (Figs 4 and 5, respectively). As indicated in Fig 4, inhibition of hypothalamic *Cbp* produces a profile of gene expression expected to decrease glycolysis. For example, inhibition of *Cbp* reduces expression of glucokinase, critical for hypothalamic sensitivity to glucose signaling via glycolysis [25]. In addition, markers for negatively regulated pyruvate dehydrogenase (e.g., increased *Pdk4* and decreased of *Pdp1*) also suggest reduced glucose utilization from glycolysis [26]. Furthermore increased expression of *G6pdx*, and *H6pd* indicates a shift in glucose metabolism away from glycolysis towards the pentose pathway [27]. Finally, increased expression of *Foxo1*, *Hif1a*, and *Txnip*, are all consistent with reduced glycolysis [7].

Inhibition of hypothalamic *Cbp* increased expression of genes promoting lipid oxidation in either mitochondria (*Cpt1a*, *Cpt1b*, *Cpt2*; Fig 5A–5C), or peroxisomes (*Crot*, *Acox3*; Fig 5D and 5E). The family of Acyl-Coa thioesterases mediates the oxidation of free fatty acids [28]. Thus, significant increases in *Acadl*, *Acads*, and *Acadvl* support that inhibition of hypothalamic *Cbp* increases fatty acid oxidation (Fig 5F–5H). Other genes potentially mediating an increase of free fatty acid metabolism include *Apod* and *Apoe*, which facilitate the release of free fatty acids from triacylglycerides [7,29] (Fig 5I and 5J).

### Enhanced expression of hypothalamic *Cpt1a* increases food intake and body weight, while reducing *Pomc* expression

**Fig 6:**
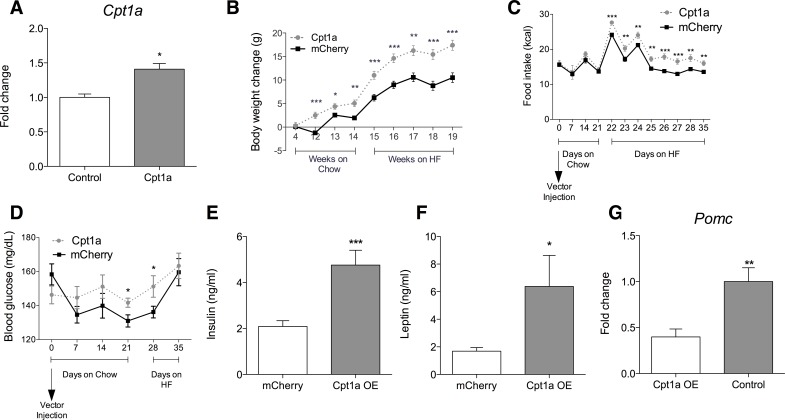
Hypothalamic *Cpt1a* overexpression increases body weight, food intake, blood glucose, insulin, and leptin, while decreasing *Pomc* expression. (A) Hypothalamic *Cpt1a* in control and overexpressing virus (n = 5–7). (B) Ad libitum body weight ± SEM measured at 4 weeks post-infusions in chow and high fat diet (*n* = 8–10). (C) Chow and high fat food intake ± SEM measured after infusions (*n* = 12–14) (D) Baseline blood glucose ± SEM measured after infusions and on different diets (*n* = 12–14). (E, F) Insulin and leptin ± SEM measured under high fat diet (*n* = 12–14). (G) Hypothalamic *Pomc* transcripts ± SEM measured by RT-PCR (*n* = 10–13). *, p < .05. **, p < .01. ***, p < .001.

The hypothalamic transcriptional profile observed in Cre-Cbp/flox suggests several mechanisms by which *Cbp* inhibition could produce obese phenotypes, including through impaired hormonal (e.g., leptin signaling) or altered nutrient signaling (e.g., switching from glycolysis to lipid metabolism). Several lines of evidence suggest that inhibiting hypothalamic ß-oxidation reduces food intake [30–33]. To assess if enhanced hypothalamic ß-oxidation would mimic effects of inhibition of *Cbp*, hypothalamic carnitine palmitoyltransferase 1A (*Cpt1a*), a rate-limiting for lipid oxidation [34], was infused in an AAV construct targeted to the ventromedial hypothalamus (VMH). Enhanced expression of *Cpt1a* (Fig 6A) increased body weight on a standard chow diet and even more on a high-fat diet (Fig 6B). Obesity was not associated with hyperphagia on the chow diet, but increased hypothalamic *Cpt1a* did produce hyperphagia on the high-fat diet (Fig 6C). Increased hypothalamic *Cpt1a* also raised baseline blood glucose before body weight gain (Fig 6D). Finally, the body weight increase after 2 weeks on high fat diet was associated with increased baseline insulin, leptin, and decreased hypothalamic *Pomc* mRNA (Fig 6E–6G).

## Discussion

We have previously reported that expression of hypothalamic *Cbp* (using the same dissection as in the present studies) positively correlates with lifespan across 5 mouse strains, and protective effects of dietary restriction during aging were prevented by inhibition of *Cbp* [8]. Nutrient sensing of metabolic status and homeostasis can be largely attributed to hypothalamic responses, and failure of such mechanisms plausibly promotes obese phenotypes [6]. Whole body *Cbp* deletion is embryonic lethal, and while *Cbp* heterozygous knockout mice are viable, these animals exhibit developmental complications resulting in distinct phenotypes such as craniofacial malformations, and decreased body weight [35]. To study the direct role of hypothalamic *Cbp* without developmental confounds, we inhibited hypothalamic *Cbp* by infusing AAV-*Cre* into adult *Cbp*-floxed mice, resulting in reduction of immunopositive cells for CBP by 74% and similar reduction in hypothalamic mRNA. Inhibition of hypothalamic *Cbp* resulted in a moderate increase in *p300* mRNA (S2 Fig), a homolog of *Cbp*, suggesting potential compensatory mechanisms [36]. As previously observed in other studies inhibiting *Cbp* by AAV-*Cre* in the adult brain [12], there was no histological or molecular evidence that this manipulation produced cytotoxicity. Similarly, inhibition of hypothalamic *Cbp* did not influence locomotion (S2 Fig), further supporting that inhibition of *Cbp* did not produce non-specific damage (e.g., leptin deficiency and hypothalamic lesions reduce locomotor behavior [37]).

Inhibition of hypothalamic *Cbp* in adults resulted in rapid and robust obese phenotypes in mice on a chow diet, including increased body weight 2 weeks after infusion of AAV-*Cre* into *Cbp*-floxed mice (Fig 2B). Inhibition of hypothalamic *Cbp* caused robust obese phenotypes independent of sex, though males were significantly heavier than females (data not shown). Weight gains were associated with robust elevation of food intake at least 3 weeks after infusion of AAV-*Cre* and persisted throughout the study (at least 5 weeks post-infusion). Decreased energy expenditure plausibly also contributed to obesity as indicated by decreased body temperature, and obesity persisted even when mice were pair-fed to controls (Fig 3B). Further studies with indirect calorimetry could clarify these mechanisms, although appropriate normalization remains a major challenge in such studies [38,39].

The obese phenotypes observed after inhibition of hypothalamic *Cbp* are plausibly mediated in part by reduced hypothalamic expression of *Pomc* and *Bdnf* (Fig 3C and 3D). Previous studies have reported that enhanced hippocampal expression of *Cbp* increases expression of *Bdnf* [40]. Hypothalamic *Pomc* expression is reduced by fasting and in genetic obesity, and transgenic enhancement of *Pomc* in leptin-deficient mice partially reverses obese phenotypes and completely corrects glucose homeostasis in these mice [41]. Conversely, feeding acutely activates Pomc neurons, an effect that may be mediated by glucose, leptin and/or insulin [2]. Similarly inhibition of hypothalamic *Bdnf* produces robust obesity, and *Bdnf* is stimulated by glucose and leptin [22]. Remarkably, inhibition of hypothalamic *Cbp* reduced expression of genes implicated in all three of the main systems that sense nutritional status (e.g., glucokinase, the leptin receptor, and insulin receptor substrate). Furthermore inhibition of *Cbp* appeared to promote a switch from hypothalamic glucose metabolism toward lipid metabolism, which could plausibly promote obese phenotypes [6]. These studies test the hypothesis that the obese and diabetic phenotypes produced by inhibition of *Cbp* may be mediated in part by induction of *Cpt1a*. Inhibition of *Cbp* induced expression of *Cpt1a* (and other genes that promote lipid oxidation), as indicated by qPCR analysis of RNA extracted from our standard ventromedial hypothalamus (VMH) dissection. Fasting (which produces pro-obesity phenotypes such as hyperphagia and reduced metabolic rate) also induces Cpt1a using the same dissection [7]. AAV-mediated enhanced expression of Cpt1a targeted to the same hypothalamic site as AAV-Cre that inhibited *Cbp* also produced obese phenotypes. Nevertheless it remains to be proven that induction of hypothalamic *Cpt1a* mediates the obese phenotypes produced by inhibition of hypothalamic *Cbp* since we did not demonstrate co-localization of the two vectors and they entailed slightly different serotypes (AAV2/8-Cre vs. AAV-2/9-Cpt1a). Nevertheless, using a protocol similar to ours, Mera et al., demonstrated that AAV-mediated delivery of the Cpt1a gene directed to the VMH of rats produced similar obese phenotypes [42], and the “AAV-infected cells in the hypothalamus were limited mainly to the VMH”. Furthermore, several studies appear to produce expression levels comparable to those we demonstrated with the AAV2/8 serotype [15,43]. In other studies directing similar AAV vectors to the VMN, transfection was also almost entirely confined to the VMN [14], possibly due to relatively impermeable interstitial tissue that prevents even small molecules from diffusing out of the VMN [4]. Nevertheless, we cannot be certain that some diffusion or retrograde transport to other areas of the brain may be responsible for some of the effects of the transfected Cre-recombinase or Cpt1a. Additionally, although *Cbp* expression is mostly restricted to neurons (particularly in the hypothalamus) in the adult murine brain [44], while similarly *Cpt1a* is found in hypothalamic neurons (although to less extent than the Cpt1c brain isoform) [42,45,46], further studies targeting glial cells are needed.

In conclusion these studies directly link hypothalamic mechanisms regulating energy balance and glucose homeostasis to mechanisms mediating effects of dietary restriction, a manipulation that is widely protective against many age-related diseases. Of particular interest, beyond important canonical mechanisms of energy balance (i.e. leptin, and insulin sensing), these mechanisms appear to share a key shift away from hypothalamic glucose metabolism and toward lipid metabolism. Finally, based on the observation that *Cbp* is a histone acetylase, we and others have demonstrated that HDAC inhibitors mimic many protective effects of dietary restriction, dependent on *Cbp* [8]. Since the ketone 3-hydroxybutyrate is an HDAC inhibitor and is produced by a ketogenic diet, these observations may explain the otherwise perplexing effects of ketogenic diets to produce weight loss [47,48] and suggests that other HDAC inhibitors may be useful to treat obesity and diabetes.

## Supporting Information

S1 Fig Virus expression as detected by GFP tag and sample of region of interest for CBP immunopositive quantification. (PDF)

S2 Fig Other changes driven by hypothalamic *Cbp* inhibition. (PDF)

S3 Fig Heat map of genes regulated by hypothalamic *Cbp* inhibition. (PDF)

S1 Table Probes and primers utilized in the study. (PDF)
