# Supplementary material for: Role of Hypothalamic Creb-Binding Protein in Obesity and Molecular Reprogramming of Metabolic Substrates
Source: PLoS One. 2016 Nov 10;11(11):e0166381. doi: 10.1371/journal.pone.0166381 (PMC5104324; doi:10.1371/journal.pone.0166381)
Supplement: S1 Fig — (PDF) [file pone.0166381.s001.pdf]

A

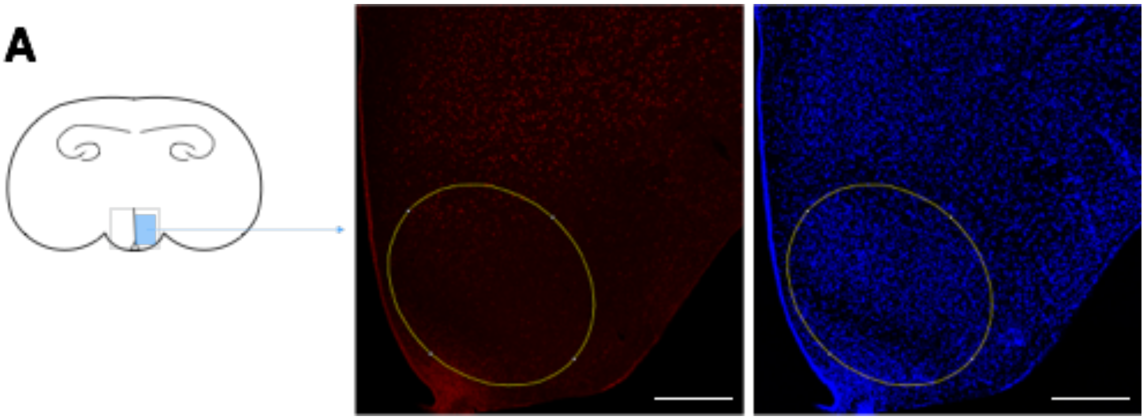

S1 Figure. Virus expression as detected by GFP tag and sample of region of interest for CBP immunopositive quantification. Representative images of coronal sections collected 3 weeks after stereotaxic delivery. Images from A-E going from rostral to caudal and corresponding Allen Atlas sections for reference. (G) Sample image (left panel Anti-CBP, right panel Hoescht) and region of interest (ROI) utilized to quantify Scale bar = 800 µm (A-F), 400 µm (G).
