# Supplementary material for: Role of Hypothalamic Creb-Binding Protein in Obesity and Molecular Reprogramming of Metabolic Substrates
Source: PLoS One. 2016 Nov 10;11(11):e0166381. doi: 10.1371/journal.pone.0166381 (PMC5104324; doi:10.1371/journal.pone.0166381)
Supplement: S2 Fig — (PDF) [file pone.0166381.s002.pdf]

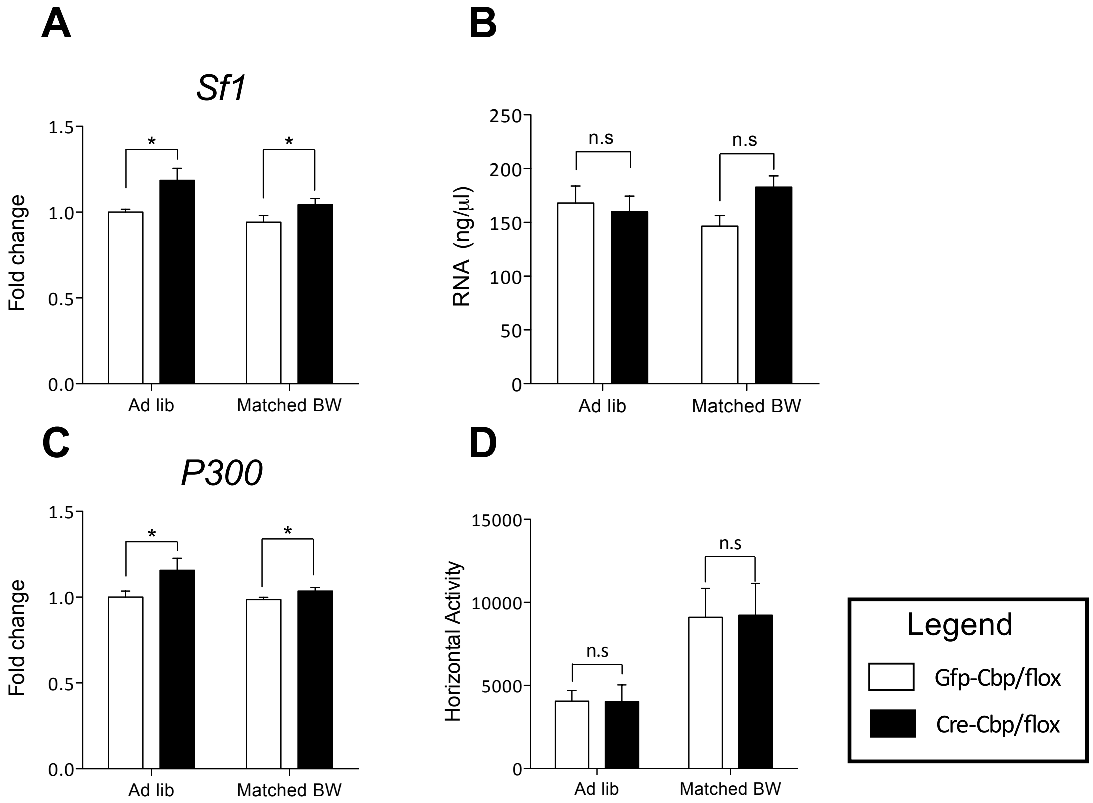

S2 Figure. Other changes driven by hypothalamic *Cbp* inhibition. Relative murine transcript abundances ± SEM as measured by nCounter, values are normalized to ad libitum control. Murine genes (A) *Sf1*, (C) *P300.* (D) RNA levels show no significant differences across groups (*n*=7-8). (d) No effect driven by the virus in horizontal activity was found in 36 hours of monitoring (*n*=4-7). *, p<.05. White bars = Gfp-Cbp/flox (controls); Solid bars= Cre-Cbp/flox (experimental).
