# Supplementary material for: Role of Hypothalamic Creb-Binding Protein in Obesity and Molecular Reprogramming of Metabolic Substrates
Source: PLoS One. 2016 Nov 10;11(11):e0166381. doi: 10.1371/journal.pone.0166381 (PMC5104324; doi:10.1371/journal.pone.0166381)
Supplement: S1 Table — (PDF) [file pone.0166381.s004.pdf]

Supplemental Table 1

Gene	NCBI Ref.	Name	Target	Sequence
Acadl	NM_007381.3	acyl-Coenzyme A dehydrogenase, long chain	641-740	GGTTAAGTGATCTCGTGATCGTCGTGGCCGTCACCAACCGTGAAGCTCGATCGCCTGCCCATGGCATTAGCCTCTTTTTGGTGGAAAACGGAATGAAAGG
Acads	NM_007383.2	acyl-Coenzyme A dehydrogenase, short chain	96-195	GTGTTCGGGACTGGCGACGGTTACACACTGTTTACCAGTCTGTGGAGCTGCCTGAGACACACCAGATGTTGCGTCAGACATGCCGTGACTTTGCCGAGAA
Acadvl	NM_017366.2	acyl-Coenzyme A dehydrogenase, very long chain	856-955	ACATTTTCACTGTCTTTGCCAAGACGCCAATTAAAGATGCAGCCACGGGGGCCGTGAAAGAGAAGATCACAGCTTTTGTAGTGGAAAGGAGCTTCGGAGG
Acox3	NM_030721.2	acyl-Coenzyme A oxidase 3	1199-1298	CCACGGATAAGGAAGAGATTCCTGTCCTTGAATATCCACTGCAGCAATGGCGCATACTTCCGTACCTGGCGGCTGCCTATGCCTTGGACCACTTCTCTAA
Apod	XM_003689301.1	apolipoprotein D	351-450	CATCTTGGGAAATGCCCGTCTCCTCCTGTGCAAGAGAATTTTGACGTGAAAAAGTATCTTGGAAGATGGTACGAAATTGAGAAGATCCCAGCGAGCTTTG
Apo3	NM_009696.2	apolipoprotein E	11-110	CGGAAGGAGCTGACTGGCCAATCACAATTGCGAAGATGAAGGCTCTGTGGGCCGTGCTGTTGGTCACATTGCTGACAGGATGCCTAGCCGAGGGAGAGCC
B2m	NM_009735.3	beta-2 microglobulin	178-277	CTGAACTGCTACGTAACACAGTTCCACCCGCCTCACATTGAAATCCAAATGCTGAAGAACGGGAAAAAAATTCCTAAAGTAGAGATGTCAGATATGTCCT
Bdh2	NM_027208.1	3-hydroxybutyrate dehydrogenase, type 2	131-230	CAGCTGCCGCTCAAGGGATTGGACGGGCATCCGCATTAGCTTTTGCAAGAGAAGGAGCCAAAGTCATAGCCACAGATATCAACGAGTCCAAACTCCAGGA
Bdnf	NM_007540.4	brain derived neurotrophic factor	3261-3360	AGTCCCGTCTGTACTTTACCCTTTGGGGTTAGAAGTCAAGTTGGAAGCCTGAATGAATGGACCCAATGAGAACTAGTGTTAAGCCCATTTCCCTAGTCAG
Cpt1a	NM_013495.1	carnitine palmitoyltransferase 1a, liver	2476-2575	CTTCCAGAAGGTTTACCGTCGGTCTCCCTAGAACAACCGTAGGCTCCACCGTTTGACTTGTGACCCTACTACATCCAGAGATGCCTTGGCTCCAGGAATA
Cpt1b	NM_009948.2	carnitine palmitoyltransferase 1b, muscle	1298-1397	ACAAGATGTCTCTGGACGCCATCGAACGTGCTGCTTTCTTTGTGACCCTGGATGAAGATTCTCATTGCTACAACCCTGACGATGAGACCAGTCTTAGCCT
Cpt2	NM_009949.2	carnitine palmitoyltransferase 2	2026-2125	GAGTTTCTCCACTGTGTCCAGAAGTGCTTGGAAGACATGTTCGATGCCCTCGAAGGCAAAGCCATCAAAACTTAGCTTCTTGGTCGATGAAAAGCCTCCA
Crot	NM_023733.3	carnitine O-octanoyltransferase	1830-1929	CCATGGTACACAATGGATATGGATTTTTCTACCACATCAGAGATGACAGGTTTGTGGTGGCATGTTCATCCTGGAGGTCATGTCCGGAGACTGATGCTGA
Foxo1	NM_019739.2	forkhead box O1	2531-2630	TTTCCTCAGACTTGGCAACAGCGGCAGCACTTTCCTGTGCAGGATGTTTGCCCAGCGTCCGCAGGTTTTGTGCTCCTGTAGATAAGGACTGTGCCATTGG
G6pdx	NM_008062.2	glucose-6-phosphate dehydrogenase X-linked	2031-2130	ACATTCTAGTTCCTGGGCTTGGACCGCCATTTTGTCCTATGCTGCTGCCACTGCCACCACCAGTAAACCCAGCTACATTCCTCAAATACCAGGCATTTAA
Gck	NM_010292.5	glucokinase (Gck), transcript variant 1	25-124	AGACATCTACTCCACACCTGGTTGGAACAGAAGCACCGACTGTGACTGAGCCCAGAGAAGAAAGCTGAGGCGTGAGGGACAGAGAGTTACCTGTTGCCTC
H6pd	NM_001291004.1	hexose-6-phosphate dehydrogenase (glucose 1-dehydrogenase)	11-110	AACATTGCAAAAAGGTTCAAAGGGGCTTTGAGTGCTCCTCCGGGCTCCCAGTTATCTTTAGAGGAGGCCGGAGAAAGGGCTCTTTGGACTTAGAGGAGAG
Hif1a	NM_010431.1	hypoxia inducible factor 1, alpha subunit	2336-2435	AGCTCATCCAAGGAGCCTTAAGCTGTCTGCCACTTTGAATCAAAGAAATACTGTTCCTGAGGAAGAATTAAACCCAAAGACAATAGCTTCGCAGAATGCT
Irs4	NM_010572.2	insulin receptor substrate 4	4869-4968	AATATGATAACAGCAGGTTGTGACCACACAGGATAGGTACAAAGGTCTTGAAGCTTGCTTAGAGCTTTGTTATGTGGTGGCACCCAGCTCTTGGATCTCG
Lept	NM_010704.2	leptin receptor	601-700	TATGTGATAGCTGCACTTAACCTGGCATATCCAATCTCTCCCTGGAAATTTAAGTTGTTTTGTGGACCACCGAACACAACCGATGACTCCTTTCTCTCAC
P300	NM_177821.6	E1A binding protein p300	4306-4405	TGGGACCTTTCTGGAGAATCGAGTGAATGACTTTCTGAGGCGACAAAATCACCCTGAATCAGGAGAGGTCACTGTTCGGGTTGTTCATGCTTCTGACAAA
Pdk4	NM_013743.2	pyruvate dehydrogenase kinase, isoenzyme 4	1356-1455	AGCGGATGACGCCTGACATTTTACGGGATCAAAGTGGGTCTGTGGCATTGCTGCTTCGTGAATGTGTGTGGACTCTAGTTTCCGCAAAACAACGCAACAC

Pdp1	NM_001098231.1	pyruvate dehyrogenase phosphatase catalytic subunit 1	121-220	AGTCTGCCACTGTTCTCTGATGCCATGCCAGCACCAACTCAACTGTTTTTTCCTCTCGTCCGTAACTGTGAACTGAGCAGAATCTATGGCACTGCATGTT
Pomc	NM_008895.3	pro-opiomelanocortin-alpha	266-365	GGAGAGCAACCTGCTGGCTTGCATCCGGGCTTGCAAACTCGACCTCTCGCTGGAGACGCCCGTGTTTCCTGGCAACGGAGATGAACAGCCCCTGACTGAA
Txnip	NM_023719.1	thioredoxin interacting protein	2341-2440	CCTGAGTGCTGCGATCAAAGGCCCAGCTTGGTTATTGCTTTTGAGGCTTTCTCCCAACGCACAGACTTGTGTAATTCTAACACTAATCCTGTGAAGGGTT

HKGs

Actinb	NM_007393.1	actin, beta, cytoplasmic	816-915	CAGGTCATCACTATTGGCAACGAGCGGTTCCGATGCCCTGAGGCTCTTTTCCAGCCTTCCTTCTTGGGTATGGAATCCTGTGGCATCCATGAAACTACAT
Hprt1	NM_013556.2	hypoxanthine guanine phosphoribosyl transferase	31-130	TGCTGAGGCGGCGAGGGAGAGCGTTGGGCTTACCTCACTGCTTTCCGGAGCGGTAGCACCTCCTCCGCCGGCTTCCTCCTCAGACCGCTTTTTGCCGCGA
Hsp90	NM_008302.3	heat shock protein 90 alpha (cytosolic)	1140-1239	AAGAAGAACAACATCAAATTGTATGTCCGCCGTGTGTTCATCATGGACAGCTGTGACGAGCTGATACCTGAGTACCTCAACTTTATCCGCGGTGTGGTTG
Ppia-ps	NM_008907.1	peptidylprolyl isomerase A	299-398	GAACTTCATCCTAAAGCATACAGGTCCTGGCATCTTGTCCATGGCAAATGCTGGACCAAACACAAACGGTTCCCAGTTTTTTATCTGCACTGCCAAGACT
Rn18s	NR_003278.3	18S ribosomal RNA	511-610	GTAGTGACGAAAAATAACAATACAGGACTCTTTCGAGGCCCTGTAATTGGAATGAGTCCACTTTAAATCCTTTAACGAGGATCCATTGGAGGGCAAGTCT

RT-PCR Primers

Gene	NCBI Ref.	Name	Direction	Sequence
Cbp	NM_001025432	Creb-binding protein	Forward	5'-GCAGCAGCAGATGAAGCAAC-3'
			Reverse	5'-GCACCTGGTTACTAAGGGATG-3'
Pomc	NM_001278582.1	Pro-opiomelanocortin-alpha	Forward	5'-GAACAGCCCCTGACTGAAAAC-3'
			Reverse	5'-AGGACCTGCTCCAAGCCTAATG-3'
