# Supplementary material for: Role of Hypothalamic Creb-Binding Protein in Obesity and Molecular Reprogramming of Metabolic Substrates
Source: PLoS One. 2016 Nov 10;11(11):e0166381. doi: 10.1371/journal.pone.0166381 (PMC5104324; doi:10.1371/journal.pone.0166381)
Supplement: S3 Fig — (PDF) [file pone.0166381.s003.pdf]

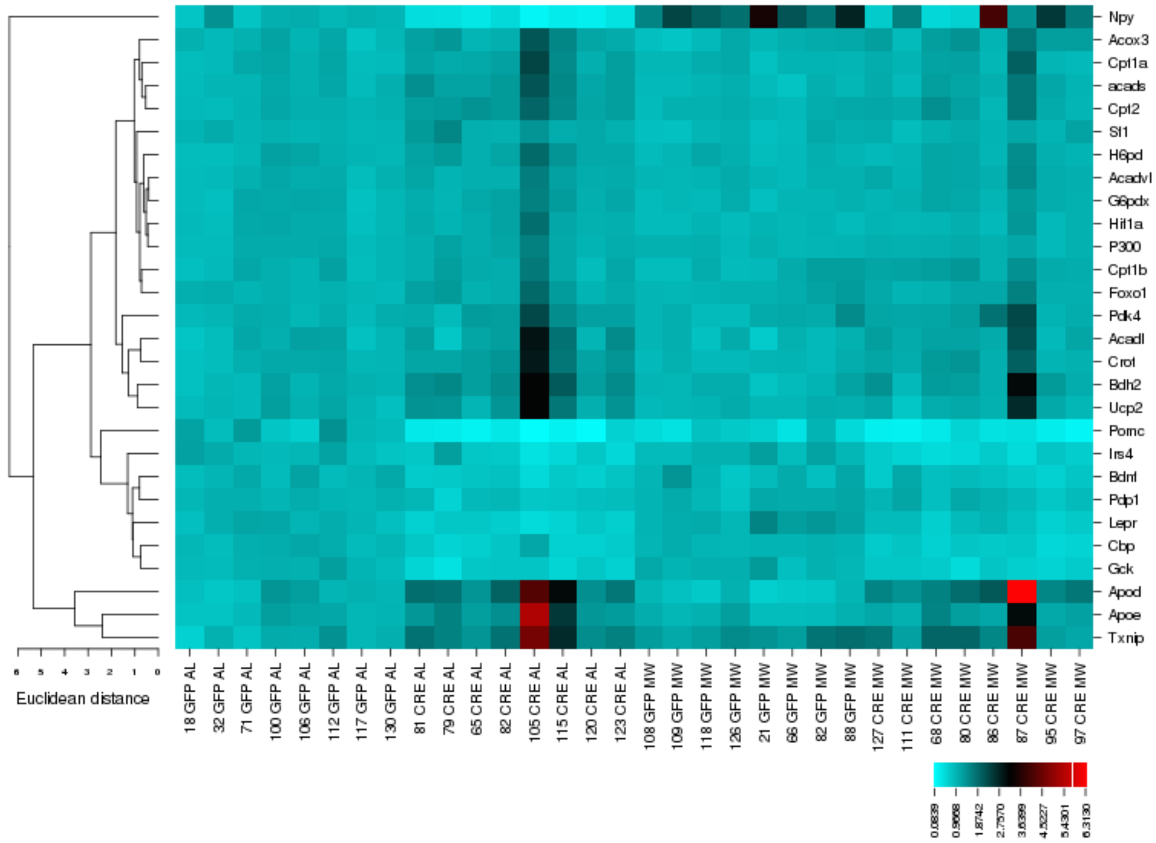

S3 Figure. Heat map of genes regulated by hypothalamic Cbp inhibition. Fold changes of transcript counts by hypothalamic *Cbp* inhibition significantly regulated by 2-way ANOVA. Genes were clustered using CIMminer.
